# Medical Psychology

**Published:** 1902-01-04

**Authors:** 


### Medical Psychology.

In these days of extreme specialisation of function, it is to be expected that each person should develop his intellect and feelings so powerfully in one direction that his vision of the world should be affected thereby. The spiritualist imagines that the phenomena of nature are controlled by unseen agencies, while the scientific man seeks for the philosopher's stone in the laws regulating the action of material atoms. To the medical psychologist the human race consists of a collection of minds more or less sane, and works of art or literature are merely a reflection of the mental condition of their originators. The result is that mental science has left its own groove and encroached upon religion, jurisprudence and art. The teaching of La Salpetrière proves conclusively that most of the witches of the Middle Ages were the unfortunate victims of neuroses who suffered from hysterical hallucinations, but to this extent only did Charcot himself believe. Other less enlightened and amateur psychologists at once endeavoured to explain all manner of obscure phenomena, such as telepathy, hypnotism, will power, and kindred manifestations of mental forces, concerning which we are at present almost entirely ignorant. Charcot himself stated "these are phenomena which only future generations will have the right to study, because the present are not ripe, not sufficiently equipped for the task." Nevertheless, the great influence of the mind over the body is considered, and allowed for far more to-day than ever before, and both physicians of repute and charlatans recognise the enormous effect of direct or implied suggestion in the treatment of certain mental diseases. The Christian Scientists and the Peculiar People dispense with all material means of treatment altogether and rely entirely upon faith. So long as this method of treatment is confined to neuroses little harm might be caused were we quite sure that neuroses really existed apart from physical, chemical, or nutritional change, but when fractures and pneumonia are treated in the same way we see at once the absurdity of the performance. But apart from enthusiastic unscientific people, the trained psychologists themselves are very apt to regard many individual peculiarities as signs of mental abnormalities, and the doctor-lawyer is now earnestly seeking to explain away crime by the hypothesis of irresponsibility. Dr. Maurice de Fleury in "Medicine and the Mind" goes so far as to state that "delinquents, criminals are actually only persons sick of will, since their will has been too feeble, too much paralysed to bridle their evil impulses" . . . . "they are the offspring of neuropaths, epileptics, drunkards or thieves; they have lived in ignorance of good and amid the contagion of evil." Undoubtedly many people who would have been punished as responsible criminals a few years ago are now treated as mental imbeciles; but it is difficult to understand, if the majority of criminals are irresponsible, how it is that crime is yearly on the decrease but insanity as steadily on the increase. In all cases of crime the motive must be steadily borne in mind, and if, no premeditated purpose can be discovered, it is conceivable that the criminal may have been seized with an uncontrollable desire; but if, for example, violence is used in order to benefit himself, by theft or otherwise, it cannot be just to society to treat such an one as an interesting invalid suffering from egomania.

Psychologists having explained away many of the cherished beliefs in religion and satisfied themselves as to the causation of crime, next devoted themselves to those sports of nature called geniuses. We do not propose here to attempt to recapitulate the various definitions of the species or to give one ourselves, but as far as we can gather the term is used by psychologists to embrace most of those people who have acquired a reputation in politics, science, art, literature, or invention. They are presumed to be quite different to the ordinary man of common sense, and to have distinct impressions of their own. Poets, artists, and fiction writers have presented a grand field for inquiry, and most of the well-known members of those professions who have suffered from sleeplessness and dyspepsia, just as does the ordinary city clerk or factory hand, have been classified and docketed as wonderful instances of neuropathy, psychopathy, and various other diseases until the pathos of the whole has long since been lost in the bathos. Since all forms of art and literature are merely the outcome of the hygiene and pathology of the intellect, Dr. Maurice de Fleury, indeed, in the work quoted above suggests that doctors should first pronounce judgment, "the mind that has dictated this is a sick mind or a healthy one, capable or not capable of contaminating those who shall read it." Now this may be an excellent idea from the point of view of the ambitious scientist, but imagine the indignation and consternation in the art world when it learnt that its works were to be submitted to a jury of medical psychologists who would probably agree that "Man is destined to study everything scientifically, to weigh everything, to measure everything; nature, the emotions of the soul, the intellectual faculties, and even works of art."

